# MPM-based simulation and bounded-error compression of material points for magnetic tactile sensors

**DOI:** 10.3389/frobt.2026.1817251

**Published:** 2026-06-15

**Authors:** Xing Lin, Guiru Lin, Ruikai Liu, Anjia Wang, Yunjiang Lou

**Affiliations:** School of Intelligence Science and Engineering, Harbin Institute of Technology, Shenzhen, China

**Keywords:** elastomer deformation simulation, point cloud compression, Real-to-Sim, robotic assembly, tactile sensor

## Abstract

Tactile sensing provides critical contact feedback for precision robotic micro-assembly, particularly in visually occluded environments common in 3C (Computer, Communication, and Consumer Electronics) manufacturing. Magnetic tactile sensors are especially promising due to their high sensitivity, fast response, and compact structure. However, the lack of effective physics-based simulation tools remains a key bottleneck for applying magnetic tactile sensing in reinforcement learning–based assembly policy training. To address this limitation, we propose a unified framework that integrates physics-based elastomer simulation, tactile-oriented point-cloud representation learning, and Real-to-Sim cross-modal mapping. This framework establishes a physically grounded intermediate representation for magnetic tactile sensing, enabling unified modeling and alignment across domains. The elastomer deformation is modeled using a particle-based formulation and simulated via the Material Point Method (MPM), achieving 0.02 mm spatial resolution with approximately 1 GB GPU memory. To enable efficient learning on high-dimensional tactile data, we propose Point-PAMAE, a masked point-cloud autoencoder with grid-based partitioning, a multi-scale dynamic graph convolutional encoder, and a position-aware decoder. The proposed method reduces partitioning overhead by 43.54% and achieves over 88% compression with a Chamfer Distance of 0.015. Furthermore, a latent-space Real-to-Sim mapping model is developed to project real magnetic signals into the simulated deformation feature space. Experimental results demonstrate that the mapped representations preserve contact-relevant geometric structures and enable reliable cross-domain tactile alignment. These results indicate that the proposed framework provides an efficient representation for magnetic tactile sensing, supporting future Sim-to-Real deployment in precision robotic assembly.

## 1 Introduction

As manufacturing systems advance toward higher precision, intelligence, and flexibility, the precision assembly of 3C (Computer, Communication, and Consumer Electronics) products has become one of the most challenging application scenarios in industrial robotics (Sun et al., 2025). Tasks such as the insertion of Flexible Printed Circuits (FPC) and Board-to-Board (BTB) connectors involve miniaturized components, fragile structures, dense pin arrays, and extremely tight tolerances. During practical assembly processes, key insertion regions are frequently visually occluded by partially pre-fixed structures, limiting the effectiveness of vision-based alignment methods (Liu et al., 2022). Although force/torque sensors can capture variations in contact loads, they lack the spatial resolution required to localize contact deformation and mating states. Consequently, tactile sensing capable of providing high-resolution contact information has emerged as a critical technological pathway for enabling automated precision assembly (Du et al., 2025; Li et al., 2025). Recent advances in flexible tactile sensing have further expanded tactile perception capabilities beyond force sensing, including multimodal perception tasks such as temperature sensing and material identification (Yao et al., 2026).

Among various tactile sensing modalities, magnetic tactile sensors perceive contact interactions by detecting changes in magnetic field distributions. Owing to their compact structure, high sensitivity, and rapid response, they demonstrate strong potential for precision manipulation in confined assembly environments (Huang et al., 2025). However, integrating tactile feedback into robotic policy learning—particularly in reinforcement learning (RL)–based autonomous assembly frameworks—remains constrained by data acquisition cost. RL relies on large-scale interaction data for policy optimization, whereas trial-and-error exploration in real precision assembly settings is inefficient and risks damaging delicate components and sensor surfaces. Consequently, simulation environments and multimodal sensing have become increasingly important for reinforcement learning–based robotic manipulation tasks such as pushing and grasping (Efendi et al., 2025).

To alleviate these limitations, Sim-to-Real policy transfer has emerged as an effective paradigm for deploying tactile-driven assembly policies (Zhao et al., 2025). This approach enables large-scale policy training in simulation environments followed by limited real-world fine-tuning, thereby balancing training efficiency and operational safety. Nevertheless, achieving stable Sim-to-Real policy transfer in tactile perception remains challenging due to two fundamental issues: (1) constructing physically realistic tactile simulation models capable of faithfully representing contact states, and (2) bridging the representation discrepancy between simulated tactile data and real sensor outputs.

Vision-based optical tactile sensors, such as GelSight, DIGIT, and OmniTact (Gomes et al., 2021; Lambeta et al., 2020; Padmanabha et al., 2020), have become widely used platforms for learning-based tactile perception and manipulation. Building on such sensors, tactile simulation research has explored rendering-based and data-driven methods for generating tactile images, especially for Sim2Real learning. More recent works have incorporated physics-based modeling, such as finite element methods (FEM) and energy-based contact formulations, to improve realism, while GPU-accelerated frameworks further enhance scalability for reinforcement learning (Zhang et al., 2025; Akinola et al., 2025). Tacchi (Chen et al., 2023) proposes a low-computational-cost elastomer deformation simulator based on the Material Point Method (MPM), enabling efficient simulation of optical tactile sensors and the generation of realistic tactile images. In parallel, data-driven approaches have been proposed to bridge the gap between simulated and real tactile signals using generative models or hybrid simulation-learning pipelines (Church et al., 2022).

Despite these advances, existing simulation frameworks, including recent physics-based approaches such as Tacchi, are primarily designed for vision-based tactile sensing and focus on image-level outputs. They do not explicitly model the physical sensing mechanism of magnetic tactile sensors, nor do they provide structured geometric representations that can support cross-modal alignment. Physics-based methods, although more accurate, often suffer from high computational cost, limiting their applicability to large-scale policy learning. Conversely, data-driven approaches improve efficiency but lack physical interpretability and generalization ability. As a result, current tactile simulation methods exhibit a fundamental trade-off between physical fidelity and computational efficiency, and remain insufficient for magnetic tactile–driven Sim-to-Real learning.

Recent efforts have begun to explore simulation for magnetic tactile sensing. For example, physics-based forward models have been proposed to describe the mapping from contact-induced deformation to magnetic signal variation (Zhou et al., 2024). While such methods provide interpretable modeling of sensing mechanisms, they mainly focus on signal prediction under simplified assumptions and do not provide structured intermediate representations suitable for learning. Consequently, they are difficult to integrate into reinforcement learning pipelines or cross-domain representation learning frameworks.

In addition to simulation, representing tactile deformation efficiently is another critical challenge. When elastomer deformation is represented as high-density point clouds, such representations provide intuitive geometric descriptions but introduce significant redundancy and computational overhead. Traditional point cloud compression methods, such as octree-based stream compression (Kammerl et al., 2012), graph-transform-based attribute coding (Zhang et al., 2014), and Gaussian-process-transform coding for point cloud attributes (de Queiroz and Chou, 2017), are designed for general point cloud compression and are not specifically designed to preserve fine-scale local deformation details critical for tactile perception. Learning-based compression and representation methods, including PointNet-based architectures (Qi et al., 2017a; Qi et al., 2017b), graph-based feature learning approaches (Wang et al., 2019), and Transformer or masked autoencoder frameworks (Yu et al., 2022; Zhao et al., 2021; Pang et al., 2023; Zhang et al., 2024), have demonstrated strong representation capability for 3D data. However, these approaches are primarily optimized for reconstruction or recognition tasks, and do not explicitly consider task-relevant local deformation structures or their role in supporting cross-modal mapping from real tactile signals. Overall, existing studies treat tactile simulation, geometric representation, and signal modeling as largely independent problems. This fragmentation limits their applicability to Sim-to-Real policy learning, where physical consistency, compact representation, and cross-domain alignment must be jointly considered. From the perspective of magnetic tactile sensing, there remains a lack of an integrated framework that simultaneously addresses physically grounded simulation, task-oriented representation learning, and Real-to-Sim feature alignment.

Motivated by these challenges, this paper proposes a unified framework integrating physical simulation, tactile-oriented representation learning, and cross-modal mapping for magnetic tactile sensing in precision assembly. First, a particle-based elastomer simulation model is constructed using the Material Point Method (MPM) to generate deformation point clouds under contact. The simulated deformation field serves as a physically grounded geometric representation tailored to the geometry, contact conditions, and cross-modal alignment requirements of magnetic tactile sensing. Second, a tactile point-cloud compression model, Point-PAMAE, is developed based on a Transformer masked autoencoder architecture, incorporating a grid-based point-cloud partitioning strategy, a multi-scale dynamic graph convolutional encoder, and a position-aware decoder to achieve high-ratio feature compression while preserving structural contact information. Finally, a latent-space Real-to-Sim tactile mapping mechanism is established to map real magnetic tactile signals into the simulated deformation feature domain, enabling cross-domain representation alignment.

The main contributions of this work are summarized as follows:

- A magnetic tactile-oriented elastomer simulation formulation is established based on MPM, achieving 0.02 mm spatial resolution while generating physically grounded deformation point clouds for subsequent feature compression and Real-to-Sim alignment.
- Point-PAMAE, a tactile-oriented masked autoencoder integrating grid-based patch generation, multi-scale dynamic graph convolution, and a position-aware decoder, is proposed, enabling over 88% feature compression while preserving deformation geometry.
- A latent-space Real-to-Sim mapping method is established to align real magnetic tactile signals with simulated deformation features, providing a representation-level basis for future downstream tactile perception and reinforcement learning–based robotic assembly tasks.

The rest of this article is organized as follows. Section II presents the overall methodology, including the physics-based tactile simulation model, the tactile-oriented point-cloud representation learning method, and the Real-to-Sim cross-modal mapping framework. Section III describes the experimental setup, including the sensor configuration, simulation environment, dataset construction, and training details. Section IV reports experimental results and analysis. Finally, Section V concludes this article.

## 2 Methodology

### 2.1 System overview and data construction pipeline

This study aims to enable efficient modeling and cross-modal mapping of magnetic tactile information for precision assembly tasks. To achieve this goal, we establish a unified framework that integrates physics-based simulation with data-driven feature learning.

The framework utilizes two primary data sources: magnetic field signals acquired from a real magnetic tactile sensor and elastomer deformation data generated through Material Point Method (MPM) simulation. By combining point-cloud feature compression and cross-modal mapping, real tactile observations can be transformed into simulated deformation representations, forming a Real-to-Sim tactile mapping pipeline.

The overall workflow consists of four stages:

Data acquisition: Magnetic field signals are collected under controlled contact interactions, covering variations in contact position, indentation depth, and pressing pose. Raw signals are preprocessed through temporal filtering, normalization, and sequence alignment to ensure data consistency.
Tactile simulation: An MPM-based elastomer simulation model is constructed to reproduce deformation responses of the elastic layer under contact. The simulation outputs high-resolution three-dimensional deformation point clouds describing surface geometry.
Feature compression: To reduce the computational cost of dense geometric data, a point-cloud autoencoder is employed to encode deformation point clouds into compact latent representations while preserving key contact structures.
4. Real-to-Sim mapping: A cross-modal mapping model is trained to learn the correspondence between magnetic signals and latent deformation features, establishing a bridge between real tactile sensing and simulated geometric responses.

Within this framework, real magnetic signals and simulated deformation point clouds are paired under aligned contact conditions to construct cross-modal tactile samples. The simulated deformation field serves as a physically grounded tactile state representation generated under matched geometric configurations, material properties, and contact conditions. Since no explicit analytical relationship exists between magnetic field variation and elastomer deformation, a learnable latent correspondence is established between real magnetic measurements and simulation-derived tactile geometry through physics-driven simulation and representation learning.

**FIGURE 1:**
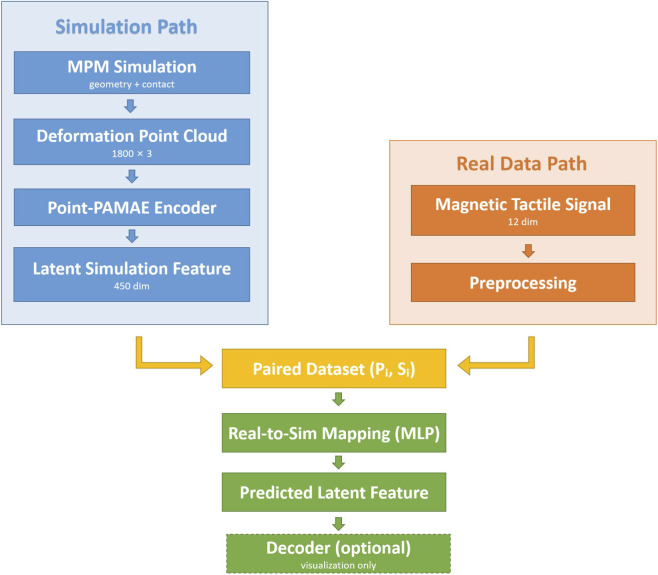
System overview and data construction pipeline of the proposed framework.

To improve the clarity of the overall methodology, a system overview and data construction pipeline is illustrated in Figure 1. The flowchart presents two parallel processing paths: a simulation pipeline and a real data pipeline. The simulation pipeline includes MPM-based elastomer deformation modeling, deformation point-cloud generation, and feature compression via Point-PAMAE, while the real data pipeline consists of magnetic tactile signal acquisition and preprocessing. These two branches are explicitly connected through paired dataset construction under aligned contact conditions. The subsequent Real-to-Sim mapping module is also illustrated, showing how real tactile signals are projected into the simulated latent feature space. In addition, key data representations at each stage, including 12-dimensional magnetic signals, 1800 × 3 deformation point clouds, and 450-dimensional latent features, are annotated to enhance clarity and reproducibility.

### 2.2 MPM-based simulation model of the elastic layer of magnetic tactile sensors

In magnetic tactile sensors, the elastic layer serves as the primary medium for perceiving contact information. Its mechanical deformation directly reflects physical interaction characteristics such as contact force distribution and contact geometry between the sensor and external objects. To faithfully reproduce the elastic response of tactile sensors during pressing interactions, the elastic layer is modeled within a continuum mechanics framework and solved using an appropriate numerical method.

Based on this requirement, an elastic-layer simulation model is constructed using the Material Point Method (MPM) to simulate contact-induced deformation between the elastomer and a rigid indenter. The simulated deformation field serves as a physically grounded geometric representation that supports tactile representation learning and subsequent Real-to-Sim cross-modal alignment.

#### 2.2.1 Simulation method and modeling requirements

The elastic layer is fabricated from soft silicone elastomer that undergoes recoverable deformation under external loads. Its behavior can therefore be described as a classical continuum mechanics problem involving kinematics, stress–strain response, and momentum conservation.

Although the Finite Element Method (FEM) provides a mature framework for elastic modeling, mesh distortion under large deformation and contact often leads to numerical instability. The Material Point Method (MPM) overcomes this limitation by combining Lagrangian material particles with an Eulerian background grid. Material points carry physical quantities such as mass, velocity, and stress, while momentum conservation equations are solved on a fixed grid at each time step. This particle–grid decoupled formulation avoids mesh entanglement and provides strong numerical robustness in contact-rich scenarios, while also supporting efficient parallel computation.

#### 2.2.2 Particle discretization and constitutive modeling of the elastic layer

The geometry of the elastic layer is constructed according to the real sensor dimensions and discretized into material particles carrying state variables including mass, position, velocity, and deformation gradient. This particle-based representation enables continuous tracking of elastomer deformation.

The elastic material is modeled as Ecoflex silicone using a fixed corotated constitutive formulation. The deformation gradient *F* is decomposed via polar decomposition to separate rigid-body rotation from pure deformation. The first Piola–Kirchhoff stress tensor P is expressed in Equation 1:

$$P\left(F\right)=2\mu \left(F-R\right)+\lambda \left(J-1\right)JF^{-T}$$ (1)

where R is the rotational component obtained from the polar decomposition of F , μ is the shear modulus, λ is the first Lamé parameter, and $J=\det\left(F\right)$ represents the volumetric deformation ratio. This formulation ensures numerical stability by eliminating non-physical stresses induced by rigid rotation.

#### 2.2.3 Numerical procedure and contact modeling

The MPM simulation adopts a particle–grid hybrid framework in which particles carry continuum states while the background grid solves momentum conservation equations. Each time step consists of initialization, Particle-to-Grid (P2G), grid update, Grid-to-Particle (G2P), and advection stages.

To enhance velocity-field representation and reduce numerical dissipation, the Affine Particle-In-Cell (APIC) scheme is employed. Information transfer between particles and grid nodes relies on interpolation kernel functions. A quadratic B-spline kernel is adopted, as shown in Equation 2:

$$N\left(x\right)=\left\{\begin{array}{cc} \frac{3}{4}-{\left|x\right|}^{2}, & 0\leq \left|x\right|<\frac{1}{2},\\ \frac{1}{2}{\left(\frac{3}{2}-\left|x\right|\right)}^{2}, & \frac{1}{2}\leq \left|x\right|<\frac{3}{2},\\ 0, & \left|x\right|\geq \frac{3}{2}. \end{array}\right.$$ (2)

The interpolation weight $w_{ip}$ between particle p and grid node i is defined in Equation 3:

$$w_{\text{ip}}=N_{i}\left(X_{p}\right)=N\left(\frac{x_{p}-x_{i}}{\Delta x}\right)N\left(\frac{y_{p}-y_{i}}{\Delta y}\right)N\left(\frac{z_{p}-z_{i}}{\Delta z}\right)$$ (3)

During the Particle-to-Grid (P2G) stage, the mass of grid node $M_{i}$ is accumulated from the contributions of particles within its influence domain, as shown in Equation 4:

$$M_{i}=\sum_{p}m_{p}N_{i}\left(x_{p}\right)=\sum_{j\in G_{i}}\sum_{p\in P_{j}}w_{\text{jp}}m_{p}$$ (4)

where $m_{p}$ and $x_{p}$ denote the mass and position of the p -th particle, respectively; $G_{i}$ represents the set of 3 × 3 × 3 grid nodes centered at node i ; and $P_{j}$ denotes the set of particles within the j -th grid cell.

Next, the momentum transfer $MG_{i}$ is computed as the sum of two components: the active momentum $MM_{i}$ and the elastic momentum $ME_{i}$ , which can be written as Equation 5:

$$MG_{i}=MM_{i}+ME_{i}$$ (5)

The active momentum term is transferred to the grid nodes through interpolation of the particles’ affine velocity fields and is explicitly expressed in Equation 6:

$$MM_{i}=\sum_{j\in G_{i}}\sum_{p\in P_{j}}w_{\text{jp}}m_{p}\left(v_{p}+C_{p}\left(X_{j}-x_{p}\right)\right)$$ (6)

The elastic momentum arises from momentum changes induced by elastic stresses. In this work, a fixed corotated elasticity model is adopted, and the first Piola–Kirchhoff stress tensor is employed. The elastic momentum term can be expressed as Equation 7:

$$ME_{i}=-\Delta t\sum_{j\in G_{i}}\sum_{p\in P_{j}}\frac{4}{\Delta X^{2}}V_{p}^{0}w_{\text{jp}}P_{p}\left(X_{j}-x_{p}\right)$$ (7)

where $V_{p}^{0}$ is the initial volume of the particle, $P_{p}$ is the first Piola–Kirchhoff stress tensor, Δt denotes the time step size, and ΔX is the grid node spacing. The grid node velocity is then obtained as the ratio of the grid node momentum to its mass, as shown in Equation 8:

$$V_{i}=\frac{MG_{i}}{M_{i}}$$ (8)

In the Grid-to-Particle (G2P) stage, particle velocities are updated via weighted interpolation of the velocities of surrounding grid nodes, as shown in Equation 9:

$$v_{p}^{\left(k+1\right)}=\sum_{i\in G_{p}}N_{i}\left(x_{p}\right)V_{p}^{\left(k\right)}=\sum_{i\in G_{p}}w_{\text{ip}}V_{p}^{\left(k\right)}$$ (9)

Meanwhile, the particle affine velocity matrix and deformation gradient are updated based on the grid velocity gradients, as shown in Equations 10, 11:

$$C_{p}^{\left(k+1\right)}=\nabla v\left(x_{p}\right)\approx \sum_{i\in G_{p}}V_{i}^{\left(k+1\right)}\nabla N_{i}\left(x_{p}\right)=\frac{4}{\Delta X^{2}}w_{jp}V_{p}^{\left(k+1\right)}\left(X_{i}-x_{p}^{\left(k\right)}\right)$$ (10)

$$F_{p}^{\left(k+1\right)}=F_{p}^{\left(k\right)}+\frac{dF_{p}}{\text{dt}}\Delta t=F_{p}^{\left(k\right)}+\nabla v\left(x_{p}\right)F_{p}\Delta t=\left(I+\Delta tC_{p}^{\left(k+1\right)}\right)F_{p}^{\left(k\right)}$$ (11)

where Δt denotes the time step size, $F_{p}$ represents the deformation gradient of particle p , $G_{p}$ is the set of 3 × 3 × 3 grid nodes surrounding particle p , and I is the identity matrix. Subsequently, particle positions are updated via advection based on the updated particle velocities, completing a full simulation time step, as shown in Equation 12.

$$x_{p}^{\left(k+1\right)}=x_{p}^{\left(k\right)}+\Delta tv_{p}^{\left(k+1\right)}$$ (12)

**FIGURE 2:**
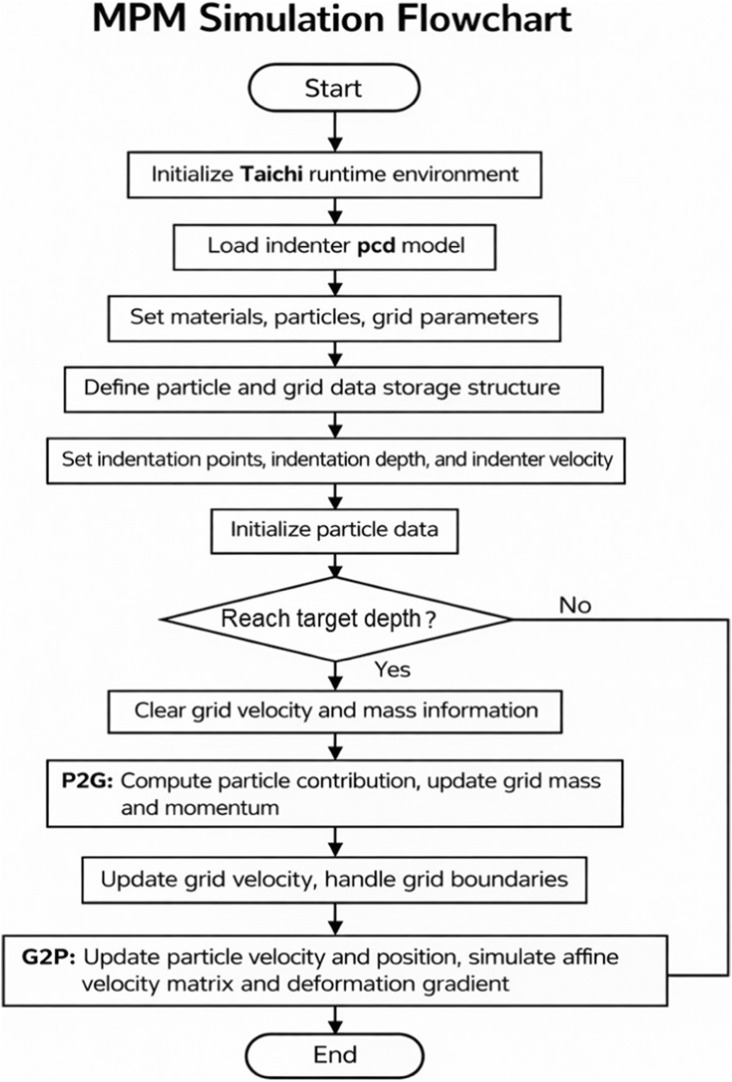
Workflow of the Material Point Method (MPM)–based elastomer deformation simulation.

For contact modeling, the indenter is treated as a rigid body whose particles move collectively under prescribed motion, while particles at the bottom of the elastic layer are fixed to represent attachment to the sensor substrate. These boundary conditions ensure physically consistent pressing interactions. The overall simulation workflow based on the MPM framework is illustrated in Figure 2.

### 2.3 Feature compression method for tactile simulation point clouds (Point-PAMAE)

Section 2.2 generates high-resolution elastomer deformation point clouds as the simulation output. While point clouds preserve fine 3D geometry, their large volume and high redundancy impose a heavy computational burden on downstream tasks (e.g., reinforcement learning). To enable efficient learning on tactile simulation data, we propose Point-PAMAE, a feature-compression method that encodes raw point clouds into compact latent representations while preserving key deformation cues.

#### 2.3.1 Baseline model: point cloud masked Autoencoder (Point-MAE)

Point-PAMAE builds upon the Point Cloud Masked Autoencoder (Point-MAE), a Transformer-based self-supervised framework. Point-MAE first partitions an input point cloud into local patches (typically via FPS + KNN), randomly masks a subset of patches with ratio $r_{\text{mask}}$ , and feeds only visible patches into a Transformer encoder. The decoder receives the encoded visible tokens together with learnable mask tokens to reconstruct the full point cloud, thereby learning transferable geometric representations.

From the perspective of data compression, if the raw point cloud is divided into N patches and the embedding dimension is D , the compressed feature space Z output by the encoder is defined in Equation 13:

$$Z=N\left(1-r_{\text{mask}}\right)\times D$$ (13)

The model’s loss function primarily guides the encoder learning by calculating the Chamfer Distance (CD) between the input point cloud X and the reconstructed point cloud Y , as shown in Equation 14:

$$d_{\text{CD}}\left(X,Y\right)=\frac{1}{\left|X\right|}\sum_{x\in X}\min_{y\in Y}{\left\|x-y\right\|}_{2}^{2}+\frac{1}{\left|Y\right|}\sum_{y\in Y}\min_{x\in X}{\left\|y-x\right\|}_{2}^{2}$$ (14)

However, directly applying Point-MAE to high-resolution tactile deformation point clouds has three limitations: (1) FPS + KNN patching is computationally expensive and produces overlapping redundancy; (2) PointNet-style patch embedding is insufficient to capture subtle indentation details; (3) explicit decoder positional encoding may leak position information, weakening representation learning.

To address these issues, we introduce three modules—GPG, MSDGC, and PAD—to improve efficiency and feature fidelity on tactile simulation data.

#### 2.3.2 Overall framework of Point-PAMAE

**FIGURE 3:**
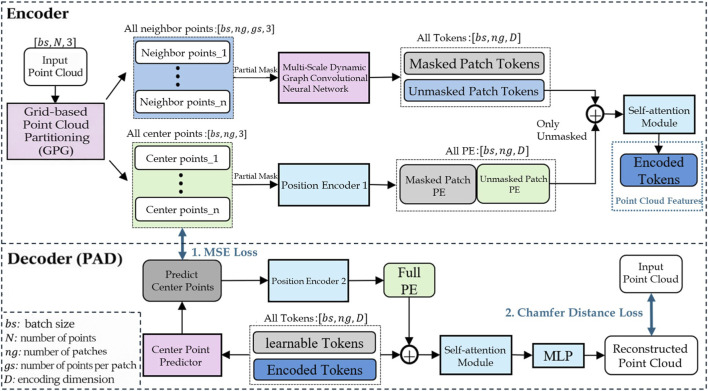
Overall architecture of the proposed Point-PAMAE tactile-oriented point-cloud compression and representation framework.

Point-PAMAE (Position-Aware Masked AutoEncoder for Point Cloud) is a self-supervised point-cloud compression framework tailored for tactile simulation. As illustrated in Figure 3, it integrates three key components:

•Grid-based Patches Generation (GPG): partitions the point cloud using grid units to reduce overlap and avoid expensive global FPS queries.
•Multi-Scale Dynamic Graph Convolution (MSDGC): extracts rich patch embeddings by leveraging local geometric relations at multiple neighborhood scales.
•Position-Aware Decoder (PAD): predicts patch centers and uses them for decoder positional encoding to mitigate positional leakage and enhance geometric representation learning.

#### 2.3.3 Grid-based Patches Generation (GPG)

##### 2.3.3.1 Point cloud voxelization

Given the raw point cloud data in Equation 15:

$$P=\;{\left\{p_{i}\in R^{3}\right\}}_{i=1}^{N}.$$ (15)

where N represents the number of points in the point cloud, and $\left(x_{i},y_{i},z_{i}\right)$ represents the 3D coordinates of each point $p_{i}$ . A voxel grid with a fixed size s is defined to map point cloud data into grid units. The grid index calculation is given in Equation 16:

$$V_{x}=\frac{x-x_{\min}}{s},V_{y}=\frac{y-y_{\min}}{s},V_{z}=\frac{z-z_{\min}}{s}$$ (16)

Where $\left(x_{\min},y_{\min},z_{\min}\right)$ are the minimum 3D coordinates of the point cloud data, s is the edge length of the grid unit, and $\left(V_{x},V_{y},V_{z}\right)$ represents the grid index to which point $p_{i}$ belongs. All points are assigned to corresponding voxel grid units $V_{mjk}$ based on their indices, as shown in Equation 17:

$$V_{mjk}=\left\{p_{i}\;\left|\;\left(V_{x}\left(p_{i}\right),V_{y}\left(p_{i}\right),V_{z}\left(p_{i}\right)\right)=\left(m,j,k\right)\right.\right\}$$ (17)

For a given grid unit $V_{\text{mjk}}$ , its surrounding 3×3×3 grid area is defined as its grid neighborhood $N\left(V_{\text{mjk}}\right)$ , and the points stored in these neighbor grids are referred to as the context point set.

##### 2.3.3.2 Center grid sampling and coverage optimization

Assuming the point cloud needs to be divided into M patches, GPG randomly selects M grids from non-empty grids as initial candidate center grids. To improve overall spatial coverage, a candidate grid replacement mechanism is introduced: the algorithm first randomly selects a candidate grid $V_{c}$ and introduces a “challenger” grid $V_{b}$ to attempt replacement. The replacement decision is jointly determined by the Coverage Gain ( $G_{\text{add}}$ ) and Coverage Loss ( $L_{\text{rmv}}$ ), as shown in Equations 18, 19:

$$G_{\text{add}}=\sum_{V\in N\left(V_{b}\right)}\delta_{g}\left(C_{V}\right)-\beta \frac{C_{V}}{\alpha }$$ (18)

$$L_{\text{rmv}}=\sum_{V\in N\left(V_{c}\right)}\delta \left(C_{V}-1\right)$$ (19)

Where $N\left(\cdot \right)$ represents the grid neighborhood, $C_{V}$ is the number of times the current grid is covered, and β is a penalty factor to avoid excessive repeated coverage (set to 0.2 in experiments). Each grid cell is allowed to be covered by at most α central grids. If $G_{\text{add}}$ > $L_{\text{rmv}}$ , the replacement is executed. This mechanism ensures that sampling centers maximize the spatial coverage of the point cloud while automatically suppressing regional overlap.

##### 2.3.3.3 Patch node sampling

After determining the center grids, point patches are constructed for each center grid from the corresponding context point set. Patch node sampling employs a hierarchical strategy: points located inside the center grid are selected first; if the quantity is less than K , required points are supplemented from adjacent grids ordered by distance. During the sampling process, repeated selection of assigned points is strictly avoided to reduce redundancy and enhance the distinctiveness between patches.

The final center *p_c_* of each patch is calculated via the mean of its K contained points, as shown in Equation 20:

$$p_{c}\left(x,y,z\right)=\frac{1}{K}\sum_{j=0}^{K}p_{j}\left(x,y,z\right)$$ (20)

##### 2.3.3.4 Complexity analysis

In the traditional “FPS + KNN” strategy, the worst-case time complexity of FPS is $O\left(N^{2}\right)$ , and KNN search has a time complexity of $O\left(\text{NM}\right)$ when partitioning patches. In contrast, in the GPG strategy: the time complexity for establishing point cloud grid indices is $O\left(N\right)$ ; the center grid sampling and coverage optimization process is $O\left(N\right)$ ; and the patch node sampling process is also $O\left(N\right)$ . Therefore, GPG significantly reduces the overall computational complexity of point cloud partitioning from $O\left(N^{2}\right)$ to approximately $O\left(N\right)$ , effectively improving the efficiency of tactile simulation point cloud data processing.

#### 2.3.4 Multi-scale dynamic graph convolutional embedding model (MSDGC)

##### 2.3.4.1 Point cloud graph convolution and edge feature extraction

PointNet-style embedding ignores local geometric relations, which are essential for capturing fine deformation patterns in tactile simulation point clouds. To address this limitation, MSDGC models each point patch as a local graph structure. For each point $p_{i}$ , its K nearest neighbors $p_{ij}$ are queried to construct edge connections. Edge features are defined using relative spatial vectors, as shown in Equation 21:

$$G=\left\{\left(p_{i},p_{ij}-p_{i}\right)|p_{i}\in P,p_{ij}\in P\right\}$$ (21)

EdgeConv is applied via an MLP on edge features and aggregated with a symmetric operator, as shown in Equations 22, 23:

$$e_{\text{ij}}=\text{ReLU}\left(\theta_{j}p_{i}+\phi_{j}\left(p_{\text{ij}}-p_{i}\right)\right)$$ (22)

$${\hat{p}}_{i}=\max_{\left(i,j\right)\in P_{ij}}e_{ij}$$ (23)

Where $\Theta =\left(\theta_{1},\theta_{2},\ldots ,\theta_{k}\right)$ and $\Phi =\left(\phi_{1},\phi_{2},\ldots ,\phi_{k}\right)$ are learnable parameters in the MLP. Here, the local features of the point cloud obtained through graph convolution operations are referred to as feature maps $\hat{P}={\left\{{\hat{p}}_{i}\in R^{D}\right\}}_{i=1}^{n}$ .

##### 2.3.4.2 Point cloud multi-scale dynamic graph convolution

To further enhance geometric representation capability, MSDGC extends graph construction to a multi-scale setting. Neighborhood graphs are dynamically updated in both Euclidean and feature spaces using different neighbor sizes (e.g., K=5,12 ), enabling the model to capture deformation cues at multiple spatial scales.

**FIGURE 4:**
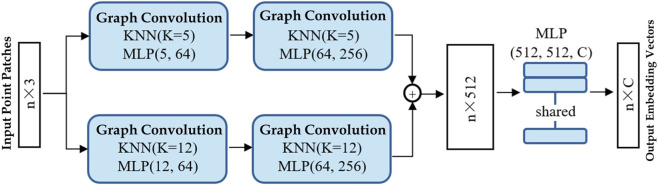
Architecture of the Multi-Scale Dynamic Graph Convolution (MSDGC) embedding model.

As shown in Figure 4, multi-scale graph features are fused along the channel dimension to produce stable and information-rich patch tokens, improving robustness to varying deformation magnitudes and point densities.

#### 2.3.5 Position-aware decoder (PAD)

In the baseline Point-MAE, decoder positional encoding is directly derived from ground-truth patch centers, which may introduce positional information leakage and reduce reconstruction difficulty.

**FIGURE 5:**
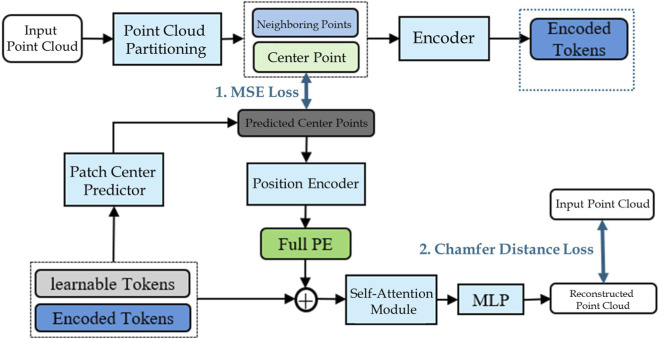
Structure of the Position-Aware Decoder (PAD) for point-cloud feature reconstruction.

To mitigate this issue, the proposed Position-Aware Decoder (PAD) incorporates a **Center Predictor** that estimates patch center coordinates—including masked patches—from encoder tokens. The predicted centers are then used for positional encoding, encouraging the encoder to learn geometry-aware structural representations rather than relying on explicit spatial priors. The structural difference between the baseline decoder and PAD is illustrated in Figure 5.

During training, the overall objective jointly optimizes center prediction and point-cloud reconstruction, as shown in Equation 24:

$$\text{loss}={\text{loss}}_{\text{MSE}}+{\text{loss}}_{\text{CD}}$$ (24)

where MSE supervises center prediction and Chamfer Distance supervises geometric reconstruction.

### 2.4 Real-to-sim cross-modal mapping

Tactile sensing plays a critical role in precision assembly scenarios where visual perception is limited by occlusion. Simulation-based reinforcement learning provides an efficient paradigm for acquiring assembly strategies; however, bridging the gap between simulated environments and real tactile sensing remains a key challenge.

To support future reinforcement learning–based robotic assembly tasks, real tactile measurements are mapped into simulation-compatible deformation representations through a Real-to-Sim cross-modal alignment framework. Leveraging the point-cloud compression model Point-PAMAE introduced in Section 2.3, the proposed method establishes a learnable correspondence between real magnetic tactile signals and simulation-derived elastomer deformation features. The resulting latent representation forms a representation-level tactile alignment framework for simulation-consistent perception and future reinforcement learning–based robotic assembly.

#### 2.4.1 Problem definition and application scenarios

In real environments, magnetic tactile sensors output low-dimensional magnetic field signals collected from multiple Hall sensing units. In contrast, simulation environments represent tactile interactions through high-dimensional elastomer deformation point clouds. These two modalities differ substantially in dimensionality, representation structure, and physical semantics, making direct cross-domain transfer difficult without an intermediate representation.

For precision assembly tasks involving small-scale 3C components, the “simulation training–real-world deployment” paradigm enables efficient policy learning. However, simulated policies rely on deformation-based tactile representations that are not directly observable from real magnetic tactile sensors. The discrepancy between simulated tactile features and real sensor measurements prevents policies from correctly interpreting real tactile inputs.

Therefore, a Real-to-Sim mapping mechanism is required to map real sensor data into simulation-compatible feature spaces. This cross-modal alignment serves as a prerequisite for representation-level Sim-to-Real transfer.

#### 2.4.2 Overall framework

The real magnetic tactile sensor comprises four Hall sensing units, each measuring three-axis magnetic flux density, forming a 12-dimensional signal vector. In contrast, simulated tactile deformation is represented by dense point clouds containing thousands of spatial points. Establishing a direct mapping between these heterogeneous modalities is challenging and prone to instability.

**FIGURE 6:**
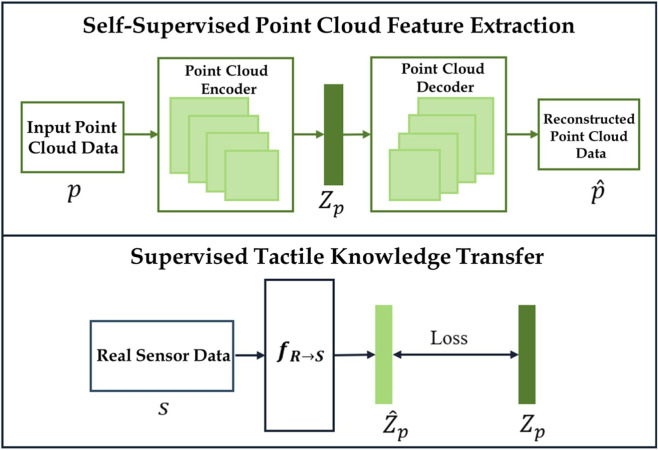
Framework of the point-cloud compression–based Real-to-Sim cross-modal mapping method.

To address this issue, we propose a two-stage Real-to-Sim cross-modal mapping framework based on point-cloud feature compression, as illustrated in Figure 6.

Stage 1: Self-supervised point-cloud feature compression. Simulated deformation point clouds are encoded using the Point-PAMAE autoencoder to obtain compact latent representations. This process compresses high-dimensional geometric data while preserving key deformation characteristics.
Stage 2: Supervised cross-modal mapping. A mapping network is trained in the latent feature space to align real magnetic tactile measurements with simulated deformation features. This design reduces dimensional disparity and enables cross-modal tactile correspondence learning.

In this formulation, simulation-derived deformation features provide structured surrogate representations under controlled and aligned contact conditions, enabling learnable correspondence modeling between real magnetic signals and simulated tactile geometry.

Through this two-stage framework, structurally heterogeneous tactile modalities are projected into a unified latent representation space, enabling representation-level cross-modal alignment and simulation-compatible tactile state representation for future reinforcement learning–based robotic assembly tasks.

#### 2.4.3 Self-supervised point cloud feature compression method

Autoencoders learn compact representations by minimizing reconstruction error while preserving structural information. As introduced in Section 2.3, Point-PAMAE is a position-aware masked point-cloud autoencoder tailored for tactile deformation data.

Within the Real-to-Sim framework, the pretrained Point-PAMAE encoder is reused as a latent feature encoder. Given simulated deformation point clouds, the encoder produces latent feature representations that serve as surrogate supervision targets for subsequent cross-modal mapping.

#### 2.4.4 Supervised real-to-sim tactile information mapping method

To model the nonlinear relationship between real magnetic signals and simulated deformation features, we employ a Multi-Layer Perceptron (MLP)-based mapping network.

Simulated point clouds are first encoded via the pretrained Point-PAMAE encoder to obtain latent representations. The Real-to-Sim mapping network then learns to regress these latent features from real tactile measurements. A two-stage training strategy is adopted. First, the point-cloud autoencoder is trained independently to ensure stable latent features. Second, the encoder is frozen and the mapping network is optimized for cross-modal alignment.

In the Real-to-Sim feature mapping phase, the training dataset consists of paired samples collected from real-world experiments and simulation environments. The dataset χ is defined in Equation 25:

$$\chi ={\left\{\left(p_{i},s_{i}\right)|p_{i}\in R^{N\times 3},s_{i}\in R^{4\times 3}\right\}}_{i=1}^{k}$$ (25)

Where k is the total number of samples, $p_{i}$ represents the simulated point cloud data, and $s_{i}$ represents the real sensor data (flattened from 4 Hall sensing units).

In the feature compression stage, the autoencoder $h\left(\cdot \right)$ is used to encode simulated point cloud data into latent feature representations $Z_{p}$ , as shown in Equation 26:

$$Z_{p}=h\left(p_{i}\right)$$ (26)

Where h represents the Point-PAMAE encoder. During Real-to-Sim training, the latent features $Z_{p}$ extracted from simulated point clouds are used as surrogate supervision targets for cross-modal alignment. These targets are derived from simulation under aligned contact conditions and should be interpreted as representation-level references. The mapping network $f_{rs}\left(\cdot \right)$ takes the real magnetic sensor signal $s_{i}$ as input and predicts the corresponding latent representation. The training objective is defined using the Mean Squared Error (MSE) loss, as shown in Equation 27:

$${\text{loss}}_{rs}=\text{MSE}\left(f_{rs}\left(s_{i}\right),Z_{p}\right)$$ (27)

To further evaluate tactile information mapping quality, the predicted latent features are decoded to reconstruct full deformation point clouds. This enables quantitative and visual assessment of Real-to-Sim mapping performance at the geometric level.

## 3 Experimental setup

### 3.1 Magnetic tactile sensor and experimental apparatus

Real tactile data were collected using a magnetic tactile sensor integrated with a six-degree-of-freedom robotic manipulator. The experimental configuration was designed to replicate practical precision assembly conditions to ensure physical validity and reproducibility.

The magnetic tactile sensor (Huang et al., 2025) was further miniaturized into a more compact structure with dimensions of 6 mm × 12 mm × 4 mm, consisting of a soft magnetic layer, a silicone elastomer layer, and a circuit layer. The soft magnetic layer contains uniformly distributed NdFeB magnetic particles, while the intermediate silicone layer provides compliant deformation space. The circuit layer integrates four three-axis Hall sensors (MLX90393) arranged in an array to measure magnetic flux density variations along the X, Y, and Z-axes.

**FIGURE 7:**
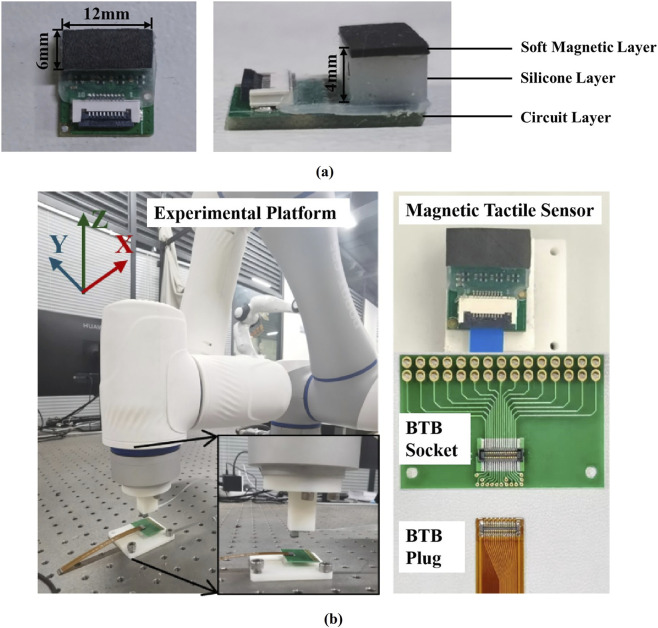
Real magnetic tactile sensor and experimental setup. **(a)** Dimensions and three-layer structure of the magnetic tactile sensor. **(b)** Real-world experimental setup of the magnetic tactile sensing system, including the robotic manipulator, the tactile sensor mounted on the end-effector, and the contact interaction during pressing.

The experimental platform employs a Dobot CR5 six-axis collaborative robot with a repeatability of 0.02 mm, enabling precise control of contact displacement and orientation. The tactile sensor (Figure 7a) is mounted on the robot end-effector to perform controlled pressing interactions. The contact object is a Panasonic AXE530127 Board-to-Board (BTB) connector plug fixed to a mating socket. By adjusting translational and rotational motions of the end-effector, diverse contact positions, indentation depths, and contact poses can be generated.

### 3.2 Simulation environment and dataset construction

The real-world experimental setup used for data collection is shown in Figure 7b, including the robotic manipulator, magnetic tactile sensor, and contact interaction during pressing tasks. Elastomer deformation simulation was conducted using the MPM-based model described in Section 2.2, with material parameters consistent with the real sensor. The elastomer geometry (6 mm × 12 mm × 4 mm) was discretized into 31 × 61 × 21 particles with a spacing of 0.02 mm. The indenter was reconstructed from the STL model of the BTB connector plug and sampled into approximately 4,000 rigid particles. The background computational grid consisted of 256 × 256 × 256 cells.

During data construction, the contact conditions are varied by adjusting indentation depth, contact area, and pressing pose. Specifically, the indentation depth ranges from 0.1 mm to 0.2 mm, the contact area varies from 25% to 100%, and the rotation angle ranges from −5° to 5°. The translational sampling step is set to 0.1 mm and the rotational sampling step is 1°, resulting in a diverse set of contact configurations for generating paired samples.

After each pressing interaction, surface particle coordinates (30 × 60) were extracted to form deformation point clouds containing 1,800 points. The final dataset comprises 162,382 paired samples, each consisting of real magnetic tactile measurements and simulated deformation point clouds under aligned contact configurations. This dataset supports subsequent feature compression and Real-to-Sim mapping experiments.

### 3.3 Training configuration and implementation details

All experiments were conducted on a workstation equipped with an NVIDIA GTX 4070 GPU. The dataset was split at the sample level into 100,000 training samples and 62,382 testing samples. Because the current study considers a single BTB connector geometry, the split was performed over paired contact samples with varying contact configurations rather than over different object instances.

For Point-PAMAE training, the Adam optimizer was used. The batch size was set to 256, the initial learning rate was set to 3e-6, the maximum learning rate was set to 1e-3, the warmup period was 10 epochs, the weight decay was 5e-2, and the total number of training epochs was 1000.

For the Real-to-Sim mapping network, an MLP with four fully connected layers was adopted. The model was optimized using Adam with an initial learning rate of 0.001 and a plateau-based learning-rate scheduler.

For feature compression and comparison experiments, Point-MAE was selected as the primary baseline, while the proposed Point-PAMAE extends it with grid-based patch generation, multi-scale dynamic graph convolution, and a position-aware decoder. Point-MAE, PCP-MAE, and Point-M2AE were trained and evaluated under identical dataset splits, preprocessing pipelines, and experimental conditions to ensure fair comparison. The main masking ratio was set to 60% for standard evaluation, while additional masking ratios of 70% and 80% were used to evaluate compression performance under higher compression levels.

In the ablation study, multiple variants were constructed, including Point-MAE, Point-MAE + PAD, Point-MAE + MSDGC, and the full Point-PAMAE model. All models were trained under identical conditions to ensure fair comparison. In addition, the number of patches and neighborhood sizes in the encoder are kept consistent across experiments unless otherwise specified. These settings ensure that the performance improvements can be attributed to the proposed modules rather than differences in training configurations.

## 4 Results and discussion

The evaluation in this study is conducted from three complementary perspectives corresponding to the objectives of the proposed framework. First, at the simulation level, the MPM-based elastomer model is evaluated in terms of deformation-geometry consistency and computational efficiency. Second, at the representation level, point-cloud compression performance is assessed using reconstruction accuracy and compression ratio, with Chamfer Distance serving as the primary geometric reconstruction metric. Third, at the cross-modal mapping level, the Real-to-Sim model is evaluated through latent-space alignment quality and the geometric consistency of reconstructed deformation point clouds. These evaluation criteria are chosen to match the representation-oriented scope of this work, rather than providing a full hardware-standard benchmarking of tactile sensor performance.

### 4.1 Validation of the elastomer simulation model

**FIGURE 8:**
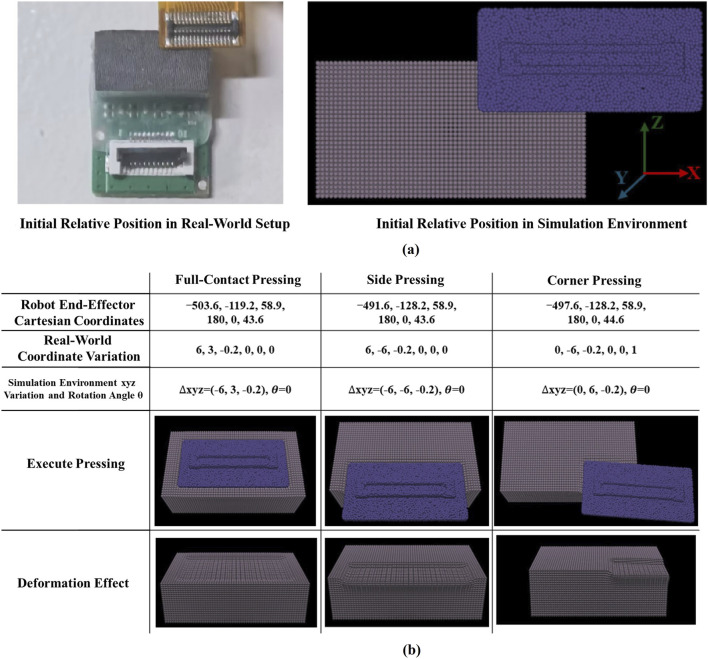
Validation of the MPM-based elastomer simulation in real and simulated environments. **(a)** Initial relative positions of the elastomer layer and indenter in the real and simulated environments. **(b)** Elastomer deformation responses under different indentation conditions, including full-contact pressing, side pressing, and corner pressing.

This section evaluates the effectiveness of the proposed MPM-based elastomer simulation model. As illustrated in Figure 8a, the initial relative positions of the elastomer layer and indenter in the real and simulated environments are shown. This setup ensures consistency between physical and simulated interactions for subsequent deformation analysis. As illustrated in Figure 8b, deformation responses of the elastic layer under different indentation conditions are visualized, including full-contact pressing, side pressing, and corner pressing.

Although minor coordinate deviations exist due to differences between the real-world and simulation reference frames, the simulation reproduces localized deformation patterns consistently across varying contact conditions. In particular, the model captures changes in indentation position, deformation extent, and boundary morphology under different pressing configurations, indicating good geometric consistency for tactile deformation modeling.

From a quantitative implementation perspective, the simulation involves over 4 × 10^5^ particles and approximately 1.6 × 10^8^ grid nodes while requiring only about 1 GB of GPU memory. Compared with typical FEM-based tactile simulation frameworks, the proposed implementation is designed to reduce computational overhead while maintaining high spatial resolution, thereby supporting efficient large-scale tactile dataset generation. The simulation is conducted at a spatial resolution of 0.02 mm, with the elastomer discretized into 31 × 61 × 21 particles and the background computational domain represented by a 256 × 256 × 256 grid. These quantitative settings support the generation of high-resolution deformation point clouds. The current evaluation focuses on geometric consistency of deformation representations under controlled contact conditions, providing representation-level validation for tactile simulation and downstream representation learning.

### 4.2 Performance evaluation of Point-PAMAE feature compression

#### 4.2.1 Comparison of partitioning strategies: GPG vs. FPS + KNN

**FIGURE 9:**
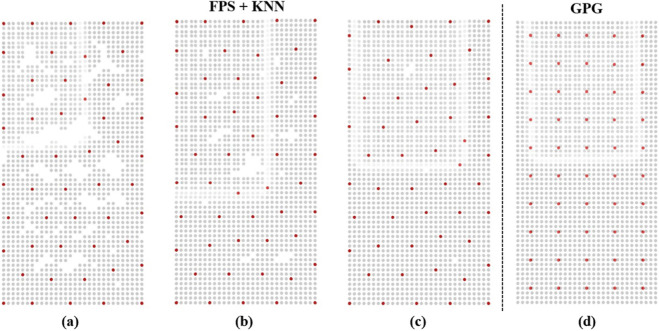
Visualization of different point-cloud partitioning strategies: **(a)** FPS + KNN with N = 50, K = 36; **(b)** FPS + KNN with N = 50, K = 50; **(c)** FPS + KNN with N = 50, K = 72; and **(d)** GPG with N = 50, K = 36. N, number of center points; K, number of neighboring points.

We compare the proposed Grid-based Patch Generation (GPG) with the conventional FPS + KNN strategy. As shown in Figure 9, FPS produces irregular and boundary-biased patch centers due to stochastic sampling, whereas GPG yields uniformly distributed grid-aligned centers, improving spatial coverage.

Under identical partition settings (N = 50), FPS + KNN fails to achieve full coverage even with K=72 , resulting in nearly 100% redundancy. In contrast, GPG partitions the cloud into structured subsets without overlap.

From a computational perspective, FPS + KNN requires 1.007 G FLOPs per sample, while GPG reduces this to 568.548 M FLOPs, achieving a 43.54% reduction. This efficiency gain stems from eliminating global KNN searches.

#### 4.2.2 Comparative analysis of model performance

**FIGURE 10:**
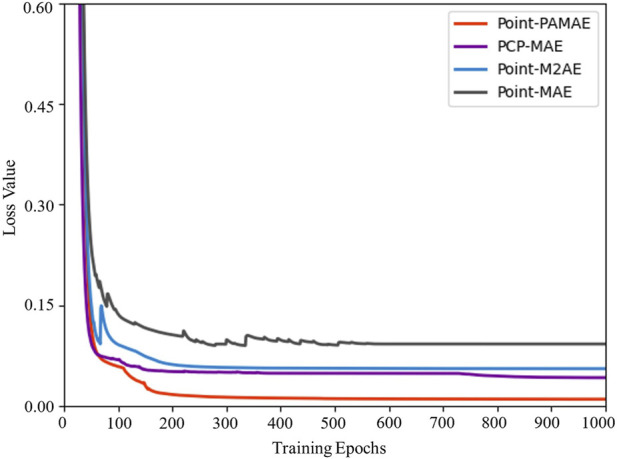
Loss curves of different point-cloud feature compression models.

**FIGURE 11:**
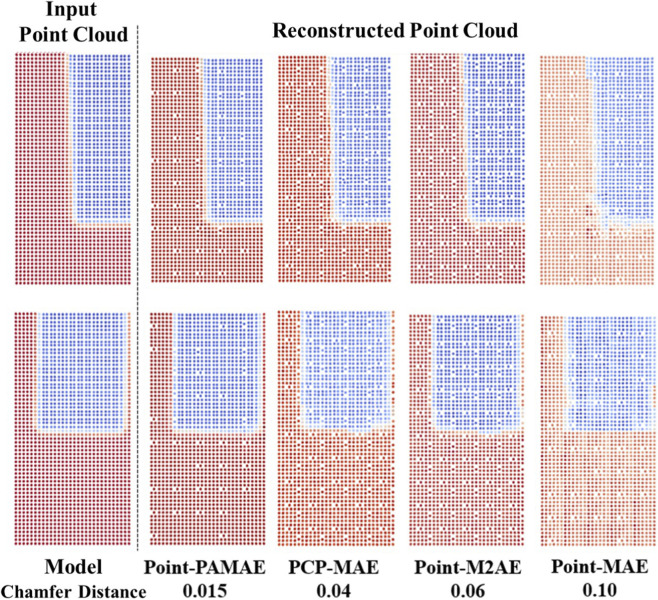
Reconstruction performance comparison across different point-cloud autoencoder models. Color variations indicate Z-axis deformation depth, where blue regions denote indentation areas.

To evaluate the feature compression capability of different models, this experiment compares Point-PAMAE with Point-MAE (Pang et al., 2023), PCP-MAE (Zhang et al., 2024), and Point-M2AE (Zhang et al., 2022). Figure 10 presents the comparison of Chamfer Distance (CD) losses for the four autoencoders, and Figure 11 displays representative point cloud reconstruction results for reference.

At a masking ratio of 60%, Point-PAMAE achieves a CD of 0.015, outperforming Point-MAE (0.10), PCP-MAE (0.04), and Point-M2AE (0.06). Reconstruction visualizations further show that Point-PAMAE more accurately preserves indentation boundaries, spatial positions, and depth morphology, suggesting improved fine-geometry representation under the present experimental setting. These results indicate that the proposed architecture provides more effective tactile deformation compression than the compared baseline models under the present experimental setting.

#### 4.2.3 Compression performance analysis

Compression performance is governed by the masking ratio. To evaluate high-compression capability, ratios of 60%–80% were tested.

As summarized in Table 1, CDs of 0.015, 0.018, and 0.022 are obtained at masking ratios of 60%, 70%, and 80%, respectively. Relative to the original 1800×3 point cloud dimension, compression ratios reach 88.8%, 91.6%, and 94.4%.

**TABLE 1:** Performance of the Point-PAMAE model in point cloud compression.

Mask ratio	Chamfer distance↓	Feature dimension	Compression ratio↑
60%	0.015	600	88.8%
70%	0.018	450	91.6%
80%	0.022	300	94.4%

**FIGURE 12:**
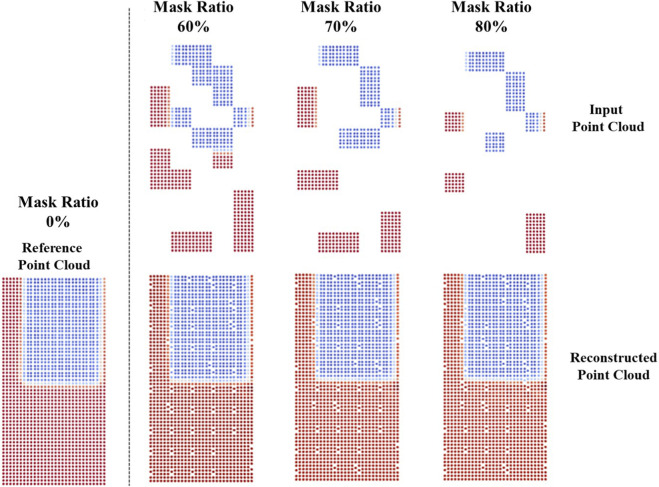
Reconstruction results under different mask ratios in Point-PAMAE.

Even under extreme compression, reconstructed point clouds preserve the global indentation morphology and spatial distribution (Figure 12), suggesting good structural retention under high compression ratios.

These results show that Point-PAMAE achieves an effective trade-off between compression efficiency and reconstruction accuracy under the present experimental setting.

#### 4.2.4 Ablation study

**FIGURE 13:**
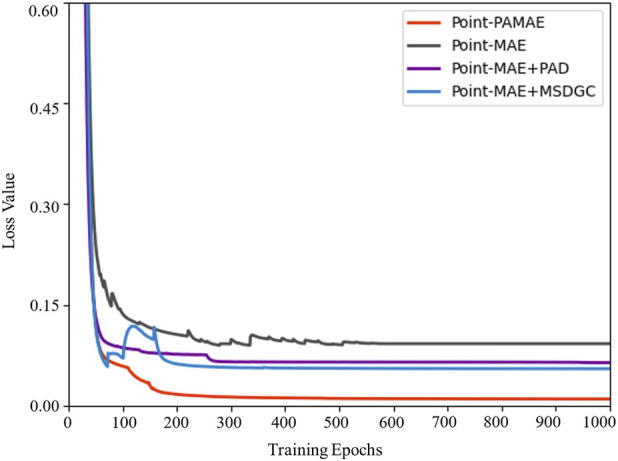
Ablation study loss curves evaluating the contributions of MSDGC and PAD modules.

Ablation experiments evaluate the contributions of MSDGC and PAD (Figure 13).

Adding PAD reduces CD from 0.10 to 0.07 (∼30% improvement), suggesting that removing explicit positional encoding improves representation learning.

Integrating MSDGC further reduces CD to 0.06 (∼40% improvement over baseline), suggesting that enhanced local geometric encoding contributes to more effective global feature compression.

### 4.3 Evaluation of real-to-sim cross-modal mapping

The Real-to-Sim mapping model is trained using paired real magnetic tactile measurements as input and simulated point-cloud features encoded by Point-PAMAE as surrogate supervision targets.

**FIGURE 14:**
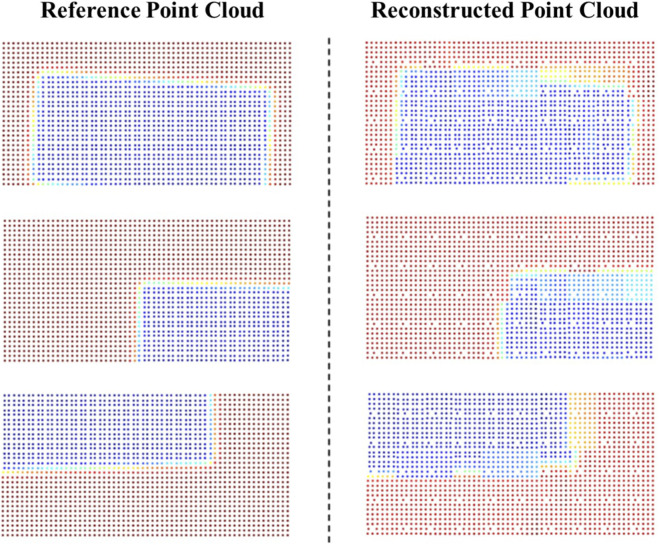
Visualization of Real-to-Sim deformation reconstruction results. The Chamfer Distance between the reconstructed and reference point clouds is 0.15.

To evaluate mapping effectiveness, predicted latent features are decoded via the Point-PAMAE decoder to reconstruct deformation point clouds. As shown in Figure 14, under identical contact conditions, the reconstructed point cloud achieves a Chamfer Distance (CD) of 0.15 compared with the corresponding simulation-derived reference point cloud. The reconstructed results preserve the main indentation geometry and spatial distribution, suggesting that the learned mapping captures meaningful correspondence between real magnetic signals and simulated tactile deformation under the present experimental setting.

To further assess the role of feature compression, Point-PAMAE is replaced with Point-MAE, PCP-MAE, and Point-M2AE under identical encoder–decoder configurations. The corresponding CDs increase to 0.20, 0.28, and 0.44, respectively, all higher than the 0.15 achieved by Point-PAMAE.

**FIGURE 15:**
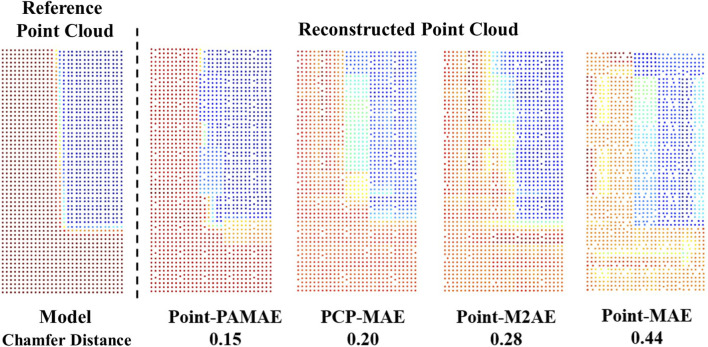
Visualization of reconstructed point clouds across different point-cloud compression models.

As illustrated in Figure 15, Point-PAMAE preserves indentation boundaries and depth morphology more effectively. These results suggest that high-quality point-cloud compression plays an important role in reducing tactile information loss during cross-modal mapping and supports more accurate contact geometry perception for assembly tasks under the present experimental setting.

### 4.4 Discussion

This study presents a unified tactile perception and mapping framework integrating elastomer simulation, point-cloud feature compression, and Real-to-Sim cross-modal mapping. Experimental results indicate that the proposed pipeline enables physically meaningful tactile representation and supports representation-level cross-modal alignment under the present experimental setting.

Regarding elastomer simulation, Section 4.1 indicates that the MPM-based model captures deformation behavior under contact loading with good geometric consistency while maintaining high computational efficiency. Compared with FEM-based approaches, the proposed model significantly reduces memory overhead (∼1 GB of GPU memory) while supporting high-resolution tactile data generation. Within the proposed framework, the simulation module functions as a physically grounded geometric representation model for magnetic tactile sensing. The resulting deformation representations provide a computationally efficient intermediate domain that supports tactile representation learning, feature compression, and Real-to-Sim cross-modal alignment.

In terms of feature representation, Section 4.2 shows that Point-PAMAE achieves improved compression and reconstruction performance under the present experimental setting. The GPG strategy improves partition uniformity and computational efficiency, while the MSDGC and PAD modules enhance local geometric encoding and reduce positional dependency. Together, these designs enable compact latent representations that preserve contact-critical deformation structures.

For cross-modal mapping, Section 4.3 suggests that a stable correspondence can be learned between real magnetic signals and simulated deformation features within the latent space. This mapping enables real tactile observations to be transformed into simulation-compatible tactile representations, allowing real and simulated tactile interactions to be interpreted within a unified latent representation space. From the perspective of reinforcement learning–based robotic assembly, the proposed framework provides an efficient tactile simulation and representation environment in which future robotic policies can be trained through large-scale interaction in simulation rather than costly real-world trial-and-error processes. This formulation also decouples tactile representation learning from downstream policy optimization. Instead of requiring reinforcement learning policies to simultaneously infer contact geometry and learn assembly strategies directly from sparse magnetic signals, the proposed framework provides a structured deformation-aware tactile representation space that is more compatible with simulation-based robotic assembly learning. Based on the proposed representation alignment framework, future deployment to real robotic systems may further require only limited real tactile data and additional adaptation strategies, such as domain alignment or online correction, to facilitate Sim-to-Real transfer for robotic assembly tasks.

Despite the promising results, several limitations remain. The current experiments primarily focus on quasi-static single-point contact conditions and do not yet cover more complex interaction scenarios such as multi-contact, sliding, or dynamic impacts. In addition, the present study does not include systematic sensitivity analysis with respect to membrane thickness, elastomer stiffness, magnetic taxel layout, indenter geometry, or combined normal–tangential loading conditions. Therefore, the current results should be interpreted as validation under controlled contact configurations for representation-level tactile modeling. In addition, although all methods were evaluated under identical experimental settings, the current study does not yet include repeated multi-seed trials, standard deviation analysis, or formal statistical significance testing. Therefore, the reported performance improvements should be interpreted as consistent empirical observations under the present experimental setup.

The proposed Real-to-Sim framework establishes a representation-level correspondence between real magnetic signals and simulation-derived deformation representations under aligned experimental conditions. Since real elastomer deformation cannot be directly measured in the current setup, the simulated deformation is interpreted as a physically grounded surrogate representation. Accordingly, the current study focuses on learning simulation-consistent tactile representations rather than recovering exact real-world deformation. Quantifying the mismatch between simulation and real deformation, as well as improving robustness against such discrepancies, remains an important direction for future work.

## 5 Conclusion

This study aims to establish a physically grounded tactile representation framework for magnetic tactile sensing that supports future reinforcement learning–based robotic assembly under limited real-world data and complex contact conditions.

To this end, we propose a unified framework that integrates MPM-based elastomer simulation, point-cloud feature compression using Point-PAMAE, and Real-to-Sim cross-modal mapping. The MPM simulation model enables physically consistent deformation generation, while the proposed Point-PAMAE architecture achieves effective compression of tactile deformation features. Furthermore, the Real-to-Sim mapping establishes a stable correspondence between real magnetic tactile signals and simulated deformation representations.

The experimental results indicate that the proposed framework can generate geometrically consistent deformation patterns, achieve improved compression performance compared with baseline methods, and enable effective cross-modal mapping between real and simulated tactile data under the present evaluation setting. These three components directly address the research objectives: (1) physically grounded tactile simulation, (2) efficient tactile data representation, and (3) cross-domain feature alignment for Sim-to-Real transfer at the representation level.

Overall, this work contributes a practical pathway for bridging real tactile sensing and simulation-based learning. Rather than directly demonstrating task-level robotic performance, the proposed framework provides a physically grounded and computationally efficient representation foundation that can support future downstream robotic assembly tasks.

Future work will focus on extending the framework to more complex interaction scenarios, including dynamic and multi-contact conditions, evaluating its performance in real-world robotic assembly tasks using task-level metrics such as success rate and efficiency, and conducting systematic sensitivity studies across sensor geometry, elastomer properties, taxel layouts, indenter shapes, and loading conditions to better assess robustness and transferability.

## Data Availability

The raw data supporting the conclusions of this article will be made available by the authors, without undue reservation.
